# CKD patients comorbid with hypertension are associated with imbalanced gut microbiome

**DOI:** 10.1016/j.isci.2025.111766

**Published:** 2025-01-09

**Authors:** Pan Wang, Yang Shen, Kaixin Yan, Siyuan Wang, Jie Jiao, Hongjie Chi, Jiuchang Zhong, Qianmei Sun, Ying Dong, Jing Li

**Affiliations:** 1 Heart Center and Beijing Key Laboratory of Hypertension, Beijing Chaoyang Hospital, Capital Medical University, Beijing, China; 2 Department of Cardiology, Beijing Chaoyang Hospital, Capital Medical University, Beijing, China; 3 Department of Nephrology, Beijing Chaoyang Hospital, Capital Medical University, Beijing, China

**Keywords:** Molecular physiology, Microbiology, Microbiome

## Abstract

Intestinal flora has been linked to chronic kidney disease (CKD) and hypertension, respectively. This study aimed to investigate the microbial community among 54 individuals without CKD, 46 hypertensive CKD patients (CKD_HTN), and 48 non-hypertensive CKD patients. Variations in microbial diversity were detected in CKD. The *Prevotella*-dominated type progressively increased from CKD to CKD_HTN. Based on the variation patterns, we identified six distinct clusters. *Klebsiella*, *Turicibacter*, and *Enterobacter* were enriched in CKD, whereas *Escherichia* and *Mogibacterium* were elevated, and *Blautia* and *Clostridium* were reduced in CKD_HTN. Enhanced phenylalanine metabolism and siderophore group nonribosomal peptides biosynthesis from non-CKD to CKD were observed, particularly in CKD with hypertension. The connections between genera and KEGG pathways suggest an impact of microbial dysbiosis on metabolism. Our findings demonstrate that imbalances in gut microorganisms and functions are associated with increased susceptibility to hypertension in CKD patients and could be targeted for improving kidney function in CKD.

## Highlights

• The gut microbial profiles in CKD patients altered further in CKD_HTN
• *Escherichia* and *Mogibacterium* elevated, *Blautia* and *Clostridium* reduced in CKD_HTN
• Gut microbial function with phenylalanine metabolism shifted in CKD_HTN patients

## Graphical abstract

**Figure:**
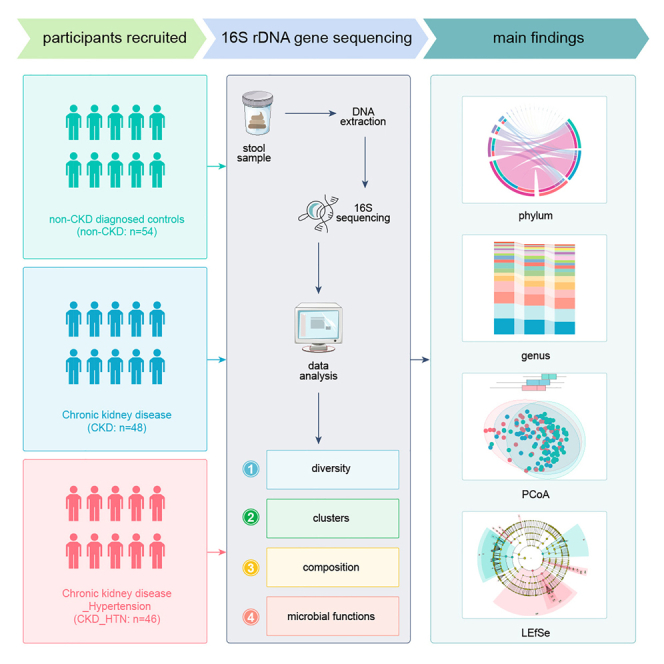

## Introduction

Kidney disease was demonstrated to contribute to significant physical limitations, loss of quality of life, and premature death.^1^ Chronic kidney disease (CKD) is estimated to affect 700–850 million populations worldwide,^2^^,^^3^^,^^4^^,^^5^ and the 2017 Global Burden of Disease Study indicated a prevalence of CKD at ∼100 million Europeans, with 300 million general population being at risk, and is projected to rank as the fifth leading cause of global death by 2040.^2^ CKD-related mortality was speculated at 1.2 million, and a further 1.4 million deaths from cardiovascular disease were suggested to be attributed to reduced kidney function.^2^ Despite these alarming figures, CKD remains one of the most neglected common chronic disease, largely due to insufficient awareness of its financial burdens and the seriously underestimated social impact.^1^

Hypertension is widely recognized as a prominent etiological factor involved in CKD pathogenesis. In CKD patients, sustained hypertension frequently accelerates the decline in kidney function, leading to high intraglomerular pressure and impaired glomerular filtration, which conversely resulted in worsening blood pressure control.^6^^,^^7^ Although aggressive control of blood pressure is associated with a reduced risk of adverse cardiovascular outcomes and mortality in the CKD patients, clinical trials have not demonstrated that intensive blood pressure management slows CKD progression.^8^ Therefore, the residual risk factors between CKD and hypertension are necessary to be explored.

The pathophysiology of hypertension in CKD is complex. Notably, accumulating evidence highlights the importance of altered host-microbiota crosstalk and gut dysbiosis, owing to its involvement in diverse diseases, including hypertension and CKD, respectively.^9^^,^^10^^,^^11^ It was demonstrated that the trillions of gut microorganisms colonizing the human gastrointestinal tract showed profound perturbation corresponding to CKD severity.^12^ Specific bacteria, such as *Ruminococcus bromii*, have been identified as varying with CKD progression.^12^ Fundamental alterations in intestinal flora have been elucidated in patients with hemodialytic end-stage renal disease via metagenomic sequencing and analysis based on metagenome-assembled genomes.^13^ Elevated levels of *Blautia* spp., *Dorea* spp., and *Eggerthellaceae*, along with depleted levels of *Prevotella* and *Roseburia* species, were observed in end-stage renal disease patients.^13^ In particular, the onset of membranous nephropathy was confirmed to depend on the presence of colonized microbiome, showing disturbed gut microbial structures and functions.^14^ Moreover, aberrant gut microbial composition and gut-derived metabolites, including trimethylamine N-oxide and short-chain fatty acids were revealed to implicate in hypertension and even preeclampsia.^15^^,^^16^^,^^17^ In addition, our previous studies have emphasized the crucial association between disordered gut microbiota, anti-hypertensive medication treatment, and hypertension development.^9^^,^^18^^,^^19^ Although the importance of gut microbial disorders has been demonstrated in CKD and hypertension separately, the role of microbiome in CKD patients susceptible to hypertension remains unclear.

Therefore, in the current study, we evaluated the gut microbial features in CKD patients with or without hypertension based on 16S rRNA gene sequencing. Our aim was to gain insights into intestinal flora dysfunction in CKD patients coupled with hypertension occurrence. The workflow of the present study is presented in Figure S1.

## Results

### General characteristics of study participants

The study cohort consisting of 54 non-CKD diagnosed controls (non-CKDs), 46 CKD patients with hypertension (CKD_HTN), and 48 non-hypertensive CKD patients was recruited in the present research (Figure S1). The baseline characteristics of participants were shown in Table S1. There was a significant difference in urine total protein (G/24H), uric acid, urine-specific gravity, eGFR, total cholesterol (TC), triglyceride (TG), and low-density lipoprotein (LDL) between the controls and CKD patients both with and without hypertension. Serum creatinine and creatinine clearance were significantly different between the control group and CKD patients with hypertension, with marginal differences between the control group and CKD patients without hypertension. No dramatic discrepancy was observed between CKD and CKD_HTN patients in terms of gender. Compared to non-CKDs and CKD, CKD_HTN patients presented with higher systolic and diastolic blood pressure (DBP) and older age. These clinical data features are associated with metabolic disturbances and water retention in the body resulting from chronic renal insufficiency.

### Hypertension occurrence in CKD patients was associated with gut microbes

**Figure 1:**
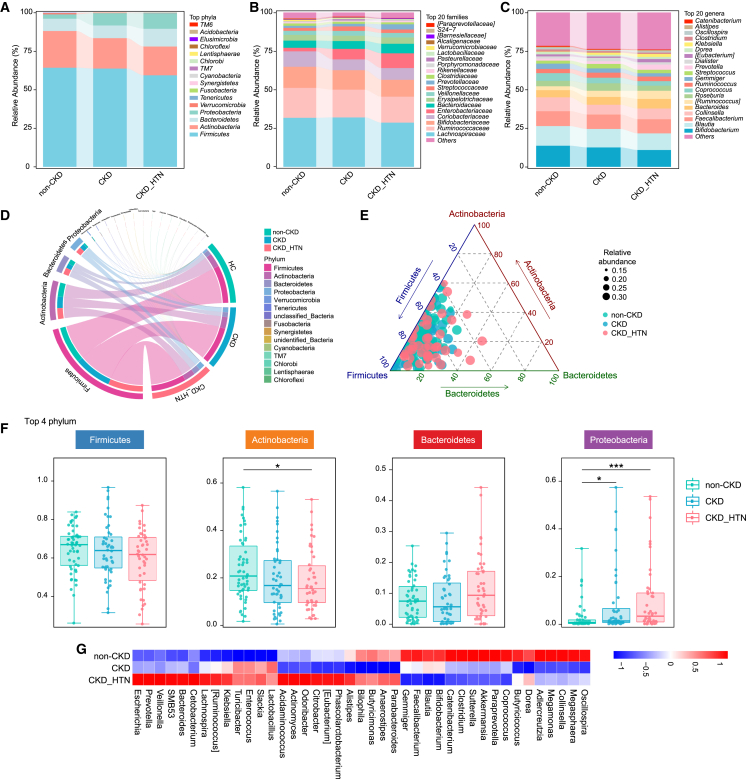
Profiles of intestinal flora in CKD patients comorbid with or without hypertension (A–C) The intestinal microbial composition of CKD patients with (CKD_HTN) or without (CKD) hypertension, and a group of non-CKD-diagnosed individuals (non-CKD) is described by the relative abundance of the most abundant phyla, families (top 20), and genera (top 20), respectively. (D) The proportion of various microbial phyla in each group is shown with Chordal graph. (E) The distribution of CKD subjects (comorbid with or without hypertension) and the controls are plotted in ternary diagram based on the relative abundance of the top three most dominant phyla (*Actinobacteria*, *Firmicutes*, and *Bacteroidetes*). (F) Comparison is conducted to determine the discrepancy in the top four phyla (*Firmicutes*, *Actinobacteria*, *Bacteroidetes*, and *Proteobacteria*) between the groups. Wilcoxon rank-sum tests (∗*p* < 0.05, ∗∗*p* < 0.01, ∗∗∗*p* < 0.001). Boxes represent the inter quartile ranges; the inside line represents the median. (G) Heatmap depicting the relative abundance and distribution of the most abundant genera across groups. The abundance profiles are transformed into Z scores by subtracting the average abundance and dividing the standard deviation of all samples. The *Z* score is negative in blue when the abundance of the row is less than the mean and positive in red when higher than the mean.

The bacterial 16S rDNA in stool samples of participants was extracted and sequenced on the Illumina platform (Figure S1). A total of 9,735,506 high-quality sequences were generated, with an average of 66,456 per sample. The total number of operational taxonomic units (OTUs) was identified to be 11,196 in non-CKD group, 8,739 in CKD patients, and 9,063 in CKD_HTN group, which was lower in CKD patients than that of controls (Figure S2A). Krona plots illustrated the relative abundance of bacterial communities at each level from phylum, class, order, and family to genus in the fecal samples of non-CKD, CKD, and CKD_HTN subjects according to taxonomic assignments of 16S rDNA gene sequences (Figure S2B). Moreover, the composition of bacterial community in different groups was further shown at the phylum, family, and genus level, respectively (Figure 1A). *Firmicutes*, *Actinobacteria*, *Bacteroidetes*, and *Proteobacteria* were the most prominent bacterial phyla across all groups, and *Lachnospiraceae*, *Ruminococcaceae*, *Bifidobacteriaceae*, *Bifidobacterium*, *Blautia*, *Faecalibacterium* etc. were observed as the 20 most abundant bacterial families and genera in the study cohort (Figures 1A–1C).

Among the non-CKD, CKD, and CKD_HTN groups, *Actinobacteria* prevailed in the controls, whereas *Bacteroidetes* and *Proteobacteria* were enriched in CKD patients with hypertension (Figure 1D). In addition, the distribution of samples from CKD patients (both with and without hypertension) and the controls based on the relative abundance of the three most dominant phyla including *Firmicutes*, *Actinobacteria*, and *Bacteroidetes* confirmed the disparities across groups (Figure 1E). Notably, for the most abundant phyla in the groups, *Actinobacteria* differed markedly between the individuals of non-CKD and CKD_HTN, showcasing a gradual reduction as CKD complication progressed (Figure 1F). Moreover, *Proteobacteria* showed a progressive increase from the CKD to CKD_HTN group compared to the controls (Figure 1F). Unfortunately, no significant differences were observed in the abundances of *Firmicutes* as well as *Bacteroidetes* among the groups. The genera detected in the subjects also exhibited disparate abundance across non-CKD, CKD, and CKD_HTN, with *Akkermansia*, *Clostridium*, *Faecalibacterium*, and *Coprococcus* simultaneously decreased, whereas *Enterococcus*, *Turicibacter*, and *Klebsiella* elevated in CKD patients both with and without hypertension (Figure 1G).

### Gut microbial diversity of CKD patients comorbid with hypertension

**Figure 2:**
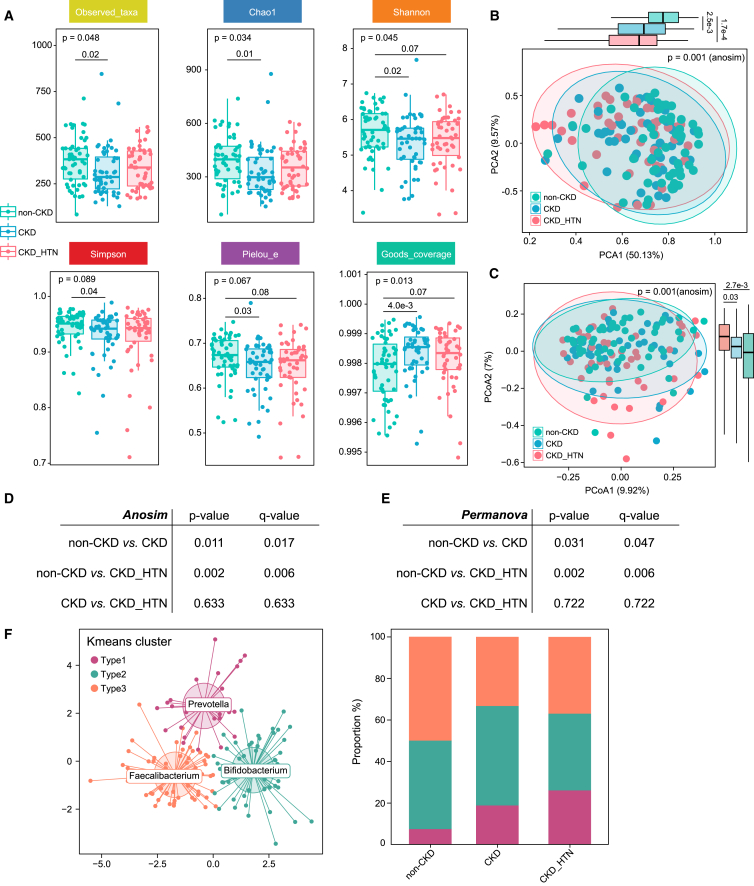
Microbial diversity and altered gut microbiota structure in CKD patients with HTN (A) Differences in alpha diversity of gut microbes among non-CKD, CKD, and CKD_HTN groups, including observed taxa, Chao1, Shannon, Simpson, Pielou, and goods coverage index. *p* values are from Wilcoxon rank-sum tests. Boxes represent the inter quartile ranges; the inside line represents the median. (B and C) PCA and PCoA distance matrix based on Bray-Curtis distance illustrate the heterogeneity of CKD patients (with and without HTN) and non-CKD-diagnosed subjects in between-habitat diversity. Different colors in the scatterplots represent samples from distinct groups. The higher the similarity between samples, the closer they distribute in the plots. Values in brackets represent the amount of total variability explained by each principal coordinate. *p* values are from the ANOSIM to test the significance of difference. Boxplot depicts the distribution of single coordinate axis in PCA1 and PCoA2. Boxes represent the inter quartile ranges; the inside line represents the median. (D and E) Comparison of beta diversity between groups is performed, and the significance of difference was determined with pairwise ANOSIM and PERMANOVA, respectively. (F) All the participants are assigned into separated clusters as identified by K-means clustering of genus-level features. The percentage of control, CKD, and CKD_HTN samples distributed in cluster 1, cluster 2, and cluster 3. There were 7.4% controls, 18.8% CKDs, and 26.1% CKD_HTNs in cluster type1; 42.6% control participants, 47.9% CKD patients, and 36.95% CKD_HTNs in cluster type2; 50% non-CKD, 33.3% CKD participants, and 36.95% patients in cluster type 3. The top genera in each cluster are labeled, with *Prevotella* prominent in cluster type 1, *Bifidobacterium* dominant in cluster type 2, and *Faecalibacterium* in cluster type 3.

To examine the variations in the gut microbial community in CKD patients with hypertension, the alpha- and beta-diversity at the genus level was analyzed based on the sequencing data. As shown in Figure S3, the rarefaction curves were tending flattened as sequencing depth advanced, reflecting the diversity of the samples and suggesting increasing the sequencing depth would not facilitate to detect more undiscovered OTUs. Compared with the non-CKD group, the CKD group showed a significantly reduced alpha-diversity of gut microbes, as evidenced by decreases in the number of taxa, Chao1, Shannon index, Simpson index, and Pielou evenness, but an increase in Goods coverage (Figure 2A), which was consistent with previous findings.^14^ Whereas, in CKD patients comorbid with hypertension, merely a slight trend toward reduced Shannon index and Pielou evenness, alongside increased Goods coverage was detected, indicating distinct degrees in disordered profile of bacterial composition for CKD patients with and without hypertension (Figure 2A).

Meanwhile, a significant difference in gut microbial beta-diversity was identified among the groups using principal-component analysis (PCA) and principal-coordinate analysis (PCoA) based on the Bray-Curtis distance (Figures 2B and 2C). The distribution of samples in PCA1 and PCoA2 illustrated more profound alterations in CKD patients coupled with hypertension occurrence. Statistic differences between groups were further assessed by analysis of similarities (ANOSIM) and permutational multivariate ANOVA (PERMANOVA) analyses, respectively (Figures 2D and 2E). It was coordinated by both methods that CKD and CKD_HTN subjects showed prominent distinction from the controls, but whether comorbid with hypertension or not exhibited no obvious difference in the CKD patients. In addition, K-means clusters were performed to cluster the 148 samples into distinct types (Figure 2F). Type 1, type2, and type3 we obtained were predominantly characterized by genus *Prevotella*, *Bifidobacterium*, and *Faecalibacterium*, respectively. The results revealed an increasing proportion of samples distributed in type 1 from non-CKD to CKD to CKD_HTN, with 7.4% controls, 18.8% CKDs, and 26.1% CKD_HTNs in type 1.

### Core gut microbial markers in CKD patients with and without hypertension

**Figure 3:**
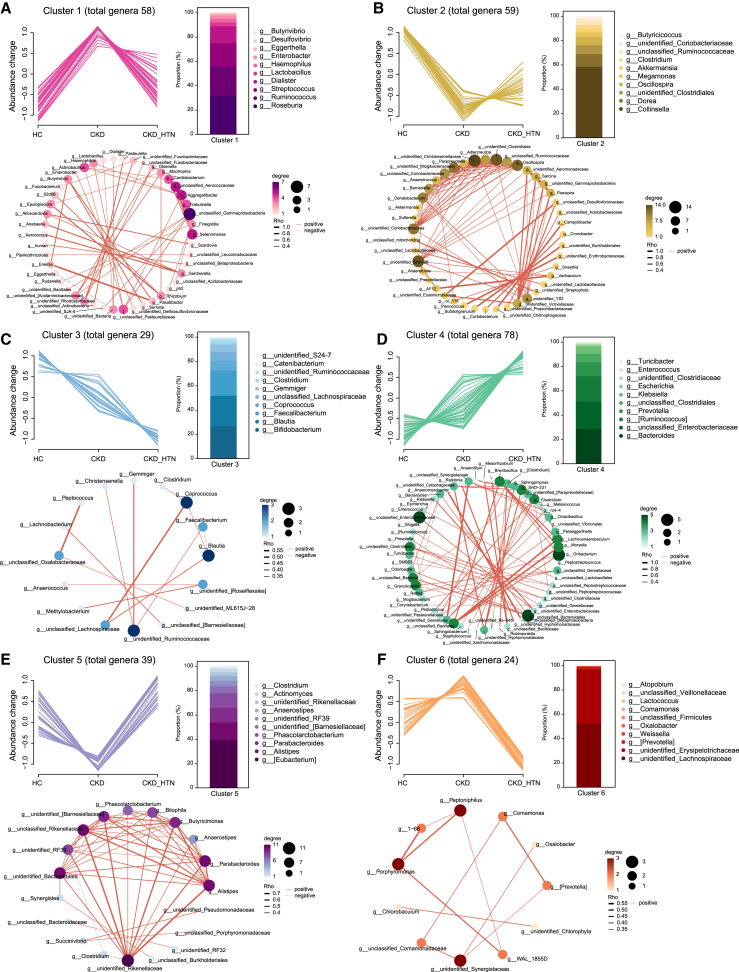
Longitudinal abundance profiles and trend patterns of intestinal microbial genera related to CKD with or without hypertension (A) Line chart of genera was obtained from Mfuzz analysis, and six clusters displayed distinct enrichment patterns from non-CKD, CKD, to CKD_HTN; 58 genera are clustered into cluster1 by their average abundances across non-CKD, CKD, and CKD_HTN groups, shown on the X axis. Abundances on the Y axis are standardized to a mean value of zero. The most abundant genus-level composition (top 10) of cluster 1 is described in bar plot. Co-occurrence network of the genera identified within cluster 1 is elucidated. The line thickness is shown according the |r| value in Spearman’s correlation; the color and size of nodes are corresponding to the linking degree. (B and F) The total number of genera included in clusters 2–6 is labeled, and abundance changes in line chart indicate the normalized level. (B) Cluster 2; (C) cluster 3; (D) cluster 4; (E) cluster 5; (F) cluster 6. Relative abundance of the most abundant (top 10) genera in differential clusters is illustrated in bar plot. The interaction network among genera in each cluster is further shown.

For the gut microbial compositions of the participants in the study cohort, Mfuzz analysis method was subsequently conducted according to the abundance changes of all the genera from non-CKD to CKD and further to CKD_HTN group (Figure 3). Based on the variation modes of distinct genera across groups, six disparate clusters were identified (Figure 3). For instance, a total of 58 genera including *Butyrivibrio*, *Desulfovibrio*, *Enterobacter*, *Lactobacillus*, *Streptococcus*, *Ruminococcus*, and *Roseburia* as the most enriched top 10 genera were determined as cluster 1 (Figure 3A). These bacteria exhibited relatively lower abundance in the control groups and elevated in CKD patients but not in CKD patients comorbid with hypertension. On the contrary, there were 59 genera in cluster 2 predominant with *Akkermansia*, *Megamonas*, and *Oscillospira*, showing depressed enrichment in CKD subjects, other than in those with hypertension (Figure 3B). Complicated interactions were observed between these genera displaying similar alterations among groups in cluster 1 and cluster 2, respectively (Figures 3A and 3B).

Moreover, it was noted that 29 genera and 78 genera, such as *Faecalibacterium*, *Blautia*, *Bifidobacterium*, *Enterococcus*, *Escherichia*, *Klebsiella*, and *Prevotella*, were included in cluster 3 and cluster 4, in which the gut microbes were progressively reduced or enhanced across groups, respectively (Figures 3C and 3D). Additionally, the gut microbes increased or decreased specifically in CKD patients with hypertension were highlighted in cluster 5 and cluster 6 (Figures 3E and 3F). Notably, *Actinomyces*, *Anaerostipes*, *Phascolarctobacterium*, *Parabacteroides*, and *Alistipes* were the most abundant genera in cluster 5, whereas *Lactococcus*, *Comamonas*, *Oxalobacter*, and *Weissella* were prominent in cluster 6. A weaker co-occurrence network was detected in cluster 3 and cluster 6, whereas strong associations were observed between those genera with similar changes in cluster 4 and cluster 5, respectively.

**Figure 4:**
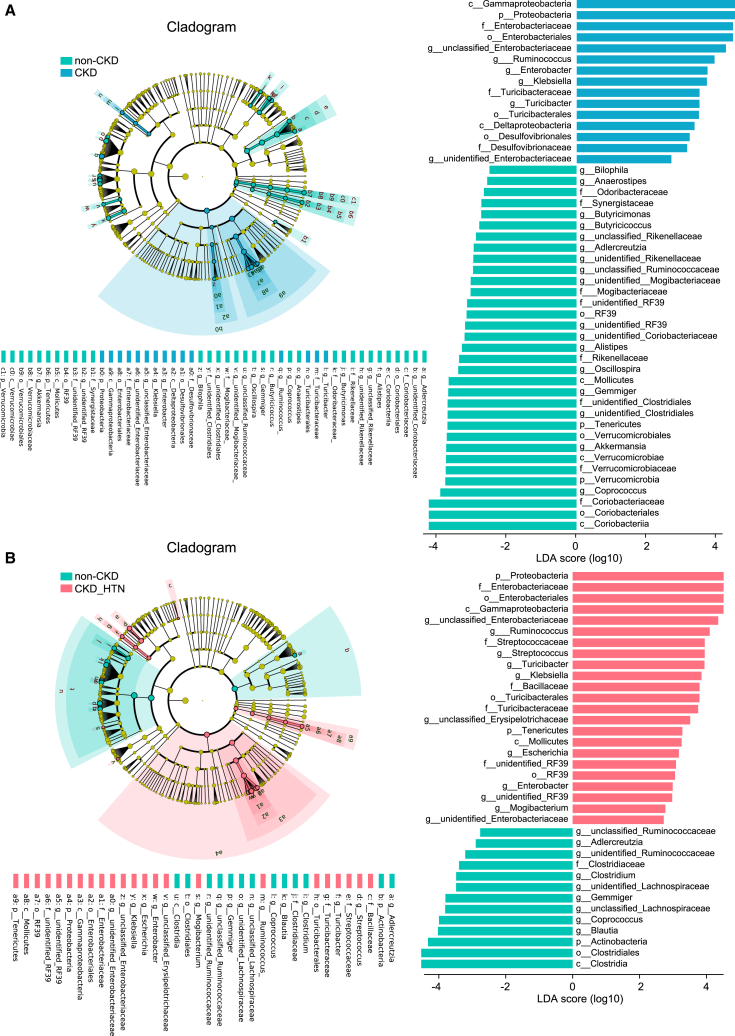
Gut bacteria and potential microbial features discriminate between CKD and HTN individuals, as well as between CKD_HTNs and non-CKDs (A) The discrepant taxonomy constitution between the CKD and non-CKD groups is revealed with LEfSe. The cladogram and bar plot of the linear discriminant analysis (LDA) score depict significantly differentially enriched taxonomic compositions in different groups. The statistical significance of different taxa is defined by LDA scores (log10) > 2 and p value <0.05. (B) Significantly different taxa between CKD_HTN and non-CKD are determined through the LEfSe analysis with bacterial abundances.

To explore the core gut microbial markers in CKD patients with and without hypertension, bacteria dramatically different as compared with the non-CKDs were discovered using linear discriminant analysis (LDA) effect size (LEfSe) analysis (Figure 4). In Figure 4A, 48 discriminant taxa, of which 15 were abundant in the CKD group and 33 were enriched in the non-CKD group were identified, such as opportunistic pathogen *Klebsiella*,^10^ the inflammation-related microorganism *Turicibacter*,^20^ and *Enterobacter* in CKD subjects, contrasted with butyrate-producing bacteria *Oscillospira*^21^ in the controls. Within the CKD patients coupled with hypertension occurrence, we found 23 significantly elevated bacteria and 13 suppressed when compared with the controls (Figure 4B). *Klebsiella*, *Turicibacter*, and *Enterobacter* were also confirmed to be enriched in CKD patients with hypertension, and we further detected increased *Escherichia* and *Mogibacterium* and reduced *Blautia* and *Clostridium* as biomarkers in CKD_HTN group. These core gut microbial markers of CKD and CKD_HTN at the genus level showed a strong correlation with clinical parameters in laboratory examination (Figure S4A). The key microbes differentially abundant between CKDs and non-CKDs and those between CKD_HTNs and non-CKDs were linked to impaired renal functions. For instance, the abundance of *Streptococcus* was positively correlated with blood urea nitrogen and proteinuria. Similarly, there was a positive correlation between *Enterobacter* and urine protein. These results suggested that gut microbial dysbiosis was involved in the hypertension occurrence of CKD patients.

### Disturbance of gut microbial functions in CKD_HTN patients

**Figure 5:**
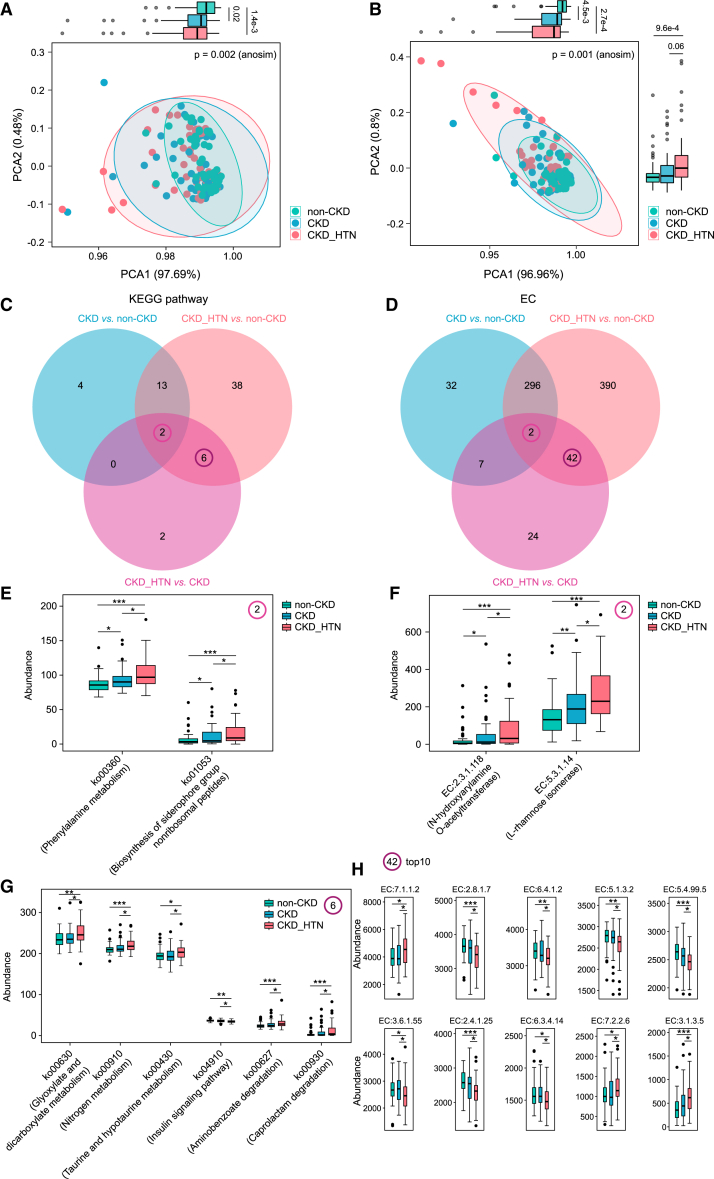
Gut microbial functions alterations in patients suffering from CKD comorbid with hypertension were summarized in KEGG pathways and ECs (A and B) Scatterplots from PCA analysis are obtained according to predicted KEGG pathway (A) and EC (B) profiles in the samples by PICRUST2. *p* values were derived from the ANOSIM test to evaluate the significance of heterogeneity between CKD patients with and without hypertension and non-CKD-diagnosed subjects. Boxplots describe the distribution of groups in principle coordinates 1 and 2, with the difference determined by Wilcoxon rank-sum test. (C and D) Venn diagram of differential microbial KEGG pathways (C) and ECs (D) altered in CKD patients vs. non-CKD controls, CKD_HTN subjects vs. non-CKD controls, and CKD_HTN subjects vs. CKDs, respectively. (E and F) Boxplots show the shared KEGG pathways (E) and ECs (F) simultaneously varied among distinct comparisons. (G) The six KEGG pathways shifted in CKD patients with hypertension as compared with both non-CKD and CKD groups. (H) Top 10 (most abundant) of the 42 ECs identified to be specifically altered in CKD patients comorbid with hypertension when compared with non-CKDs and CKDs. Boxes represent the inter quartile ranges; the inside line represents the median.

According to the Kyoto Encyclopedia of Genes and Genomes (KEGG) database, we evaluated gut microbial functions among groups, annotating 174 KEGG pathways and 2,110 enzymes (ECs). PCA based on KEGG pathways and ECs distinguished CKD patients from controls, and ANOSIM analysis further confirmed the differences among groups (Figures 5A and 5B). The distribution of samples in PCA1 or PCA2 indicated more profound dysbiosis of microbial functions in CKD patients with hypertension. A total of 65 KEGG pathways and 793 ECs were dramatically different between groups, as determined by the Wilcoxon rank-sum test (Figures 5C and 5D). Notably, it was observed that 2 KEGG pathways and 2 ECs were found to be synchronously altered when compared was conducted between groups. The relative abundances of the differential KEGG pathways (ko00360 and ko01053) and ECs (EC:2.3.1.118 and EC:5.3.1.14) shared in the comparison are presented in Figures 5E and 5F. Intriguingly, these KEGG pathways and ECs we focused on, known as phenylalanine metabolism, biosynthesis of siderophore group nonribosomal peptides, and N-hydroxyarylamine O-acetyltransferase, L-rhamnose isomerase, elevated asymptotically from non-CKD status to CKD disease state, and further in CKD complicated with hypertension.

In addition, we identified another 6 KEGG pathways and 42 ECs significantly altered in CKD patients with hypertension as compared with both non-CKD and CKD groups, which were described in Figures 5G, 5H, and S5A. The microbial capacities in glyoxylate and dicarboxylate metabolism (ko00630), nitrogen metabolism (ko00910), taurine and hypotaurine metabolism (ko00430), aminobenzoate degradation (ko00627), and caprolactam degradation (ko00930) were enhanced when CKD occurred coupled with hypertension (Figure 5G). A series of ECs, such as EC:2.8.1.7, EC:6.4.1.2, EC:5.1.3.2, EC:5.4.99.5, EC:3.6.1.55, EC:2.4.1.25 and EC:6.3.4.14 were found to reduce dramatically in CKD_HTN group, whereas EC:7.1.1.27.1.1.2, EC:7.2.2.67.2.2.6, EC:3.1.3.5, EC:1.16.3.2, EC:1.2.5.1, and EC:1.4.1.3 were increased (Figures 5H and S5A).

**Figure 6:**
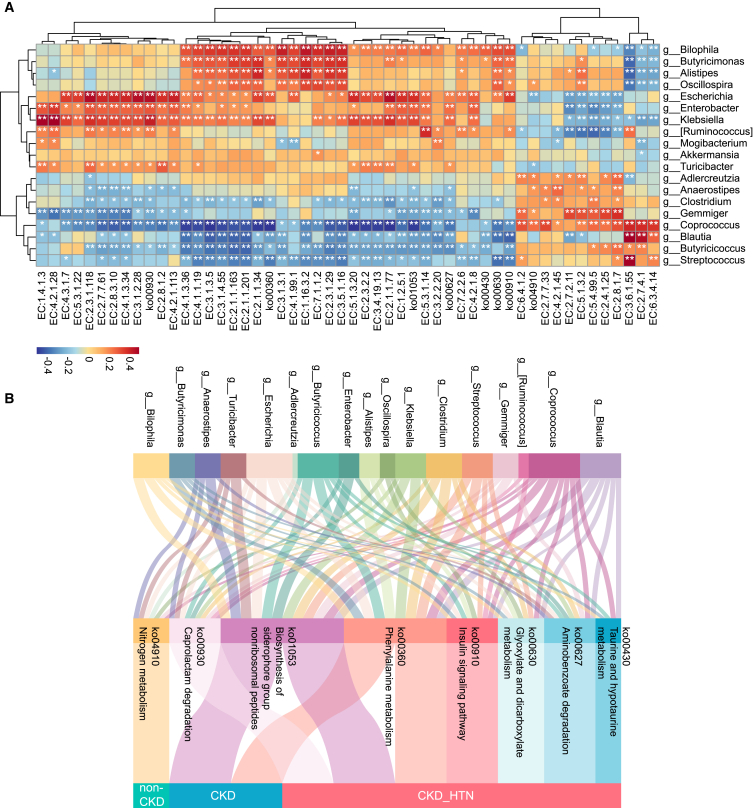
Correlation of the microbial core genera, ECs, and KOs in CKD patients with hypertension (A) The key genera discriminate between CKD and non-CKD individuals and between CKD_HTNs and non-CKDs are listed, and the potential association among these key genera and core ECs/KOs (identified in Figures 5E–5H) is determined with Spearman’s correlation analysis. Heatmap describes the positive (red) or negative (blue) associations according to |r|, and statistic difference is labeled with ∗*p* < 0.05 and ∗∗*p* < 0.01. (B) Sankey plot showing the linkages of key genera, the crucial KEGG pathways in CKD_HTN, and the distribution of differential KEGG pathways involved in CKD.

Furthermore, Spearman’s correlation analysis between the significantly distinct gut microbial functions and discriminant genera identified by LEfSe analysis was performed. Core gut microbes showing differential enrichment between CKDs and non-CKDs, as well as between CKD_HTNs and non-CKDs, were observed to be strongly associated with the KEGG pathways and ECs (Figure 6A). It was suggested that the genera such as *Enterobacter* and *Klebsiella* enriched in the CKD and CKD_HTN group were positively correlated with ko00627, ko00930, ko01053, and a majority of ECs, including EC:2.7.7.61, EC:2.8.3.10, EC:4.1.3.34, EC:3.1.4.55, and EC:2.1.1.163 *Escherichia*, a biomarker in CKD_HTN group, showed relationships with the pathways and ECs similar to *Enterobacter* and *Klebsiella*, whereas *Blautia* and *Clostridium*, which were specifically depressed in CKD patients with hypertension, exhibited a negative correlation with ko00627, ko01053, and EC:2.7.7.61, EC:2.8.3.10, EC:4.1.3.34, EC:3.1.4.55, and EC:2.1.1.163. These key genera displayed complicated linkages to KEGG pathways among the controls and CKD patients, especially in those with hypertension, revealing a potential influence of gut microbial dysbiosis on metabolism (Figure 6B).

## Discussion

In the present study, a comprehensive analysis was conducted using 16S rRNA gene sequencing to investigate the altered microbial community in non-CKD diagnosed controls, CKD patients, and CKD_HTN patients. Our findings revealed a distinct gut microbiome signature in CKD patients with or without hypertension. Notably, there was a progressive shift toward a Prevotella-dominated microbial type from CKD to CKD_HTN patients compared to non-CKD controls. Additionally, we observed gradual increases in gut microbial functions related to phenylalanine metabolism and biosynthesis of siderophore group nonribosomal peptides from non-CKD controls to CKD and then to CKD_HTN. These perturbations in gut microbiota, along with imbalanced KEGG pathways and ECs (enzymatic reactions), are associated with the occurrence of CKD in hypertension.

The prevalence of hypertension gradually increases across CKD stages, making it the most common comorbidity in CKD patients. It is worth noting that approximately 90% of end-stage renal disease patients suffer from hypertension.^22^ Recent researches have underscored the significance of gut microbial dysbiosis as a pivotal factor in the pathophysiology of both CKD and hypertension^9^^,^^12^ individually, presenting it as a promising avenue for early diagnosis and treatment. Nonetheless, the gut microbial composition and functionality in CKD patients with hypertension have been significantly overlooked. In this study, we have identified a distinctive signature of gut microbiome consisting of differentially abundant microbial genera in CKD and CKD_HTN, respectively, when compared to non-CKD controls. The findings of various studies have indicated that the depletion of butyrate-producing bacteria, such as *Coprococcus* and *Faecalibacterium*, in patients with CKD may exacerbate the progression of CKD by impairing the function of the intestinal barrier and triggering excessive inflammation.^23^^,^^24^^,^^25^ Also, the decrease in the abundance of *Coprococcus* and *Faecalibacterium* has been observed in patients with pre-hypertension and hypertension.^10^^,^^26^ Consistent with previous research, we observed a significant reduction in the abundance of *Coprococcus* genera among CKD patients. Furthermore, both *Coprococcus* and *Faecalibacterium* exhibited a progressive decrease from the CKD to CKD_HTN group when compared to controls. Additionally, there was a positive correlation between DBP, serum creatinine levels, and the abundance of *Coprococcus* at the genus level.

Meanwhile, *Prevotella*-dominated microbial type and specific microbial markers such as *Klebsiella* and *Turicibacter* in CKD_HTN patients is a major finding. *Prevotella*, known for its involvement in carbohydrate and protein fermentation, has been associated with metabolic disorders, especially as a prominent gut enterotype in hypertension.^9^^,^^27^ In CKD patients, the accumulation of uremic toxins and altered metabolic pathways may serve as a favorable environment for the growth of *Prevotella*, leading to its dominance in the gut microbiome.^28^ This overrepresentation might further exacerbate inflammation and metabolic imbalances, thereby contributing to the progression of hypertension in these patients.

*Klebsiella* is a known opportunistic pathogen that has been linked to gut dysbiosis and increased intestinal permeability in hypertensive subjects.^10^ The *Klebsiella* is capable of producing a range of uremic toxins indirectly, including lipopolysaccharide, indoxyl sulfate, and p-cresyl sulfate, which can serve as reliable indicators for the progression of CKD.^29^ In the context of CKD and hypertension, the impaired renal function and altered gut environment may further facilitate the colonization and proliferation of *Klebsiella*, potentially leading to adverse health outcomes. Indeed, previously, it has been observed that *Klebsiella* in the gut environment are significantly more abundant in patients with CKD, showing a progressive increase as the severity of CKD escalates in canines.^30^ Consistently, a receiver operating characteristic (ROC) curve generated using the relative abundance of *Klebsiella pneumoniae* demonstrated a gradual increase with increasing CKD severity, suggesting its etiological importance in the deterioration of CKD patients.^31^ Furthermore, compelling evidence suggests that *Klebsiella pneumoniae* is implicated in a substantial proportion of deaths (26%) and heart-disease-related deaths (51%) among individuals with CKD.^32^ The causal relationship between *Klebsiella pneumoniae* and hypertension development has also been established.^10^ Collectively, these robust findings strongly implicate *Klebsiella* in the progression of CKD, particularly among patients with comorbid hypertension, thereby providing a compelling rationale for further investigation into this potential causal pathway.

In addition, *Turicibacter* has been implicated in gut microbial imbalances and associated with various disease states.^33^^,^^34^^,^^35^ Previous study indicated that *Turicibacter* is associated with circulating inflammatory markers such as interleukin-1β (IL-1β), and that reducing its expression level can maintain gut microbiome homeostasis.^20^^,^^26^ Consistent with our findings, Wang et al. reported a significant elevation in the abundance of the microbial species *Turicibacter sanguinis* among patients diagnosed with end-stage renal disease.^12^ Additionally, the quantity of *Turicibacter* was found to be significantly higher in spontaneously hypertensive rats, an animal model of hypertension, compared to their corresponding controls.^36^ The presence of these specific microbial markers in CKD patients with hypertension may reflect an overall disruption of the gut microbiome and an interplay with host metabolism. In summary, the identification of *Prevotella*-dominated microbial type and specific microbial markers such as *Klebsiella* and *Turicibacter* in CKD patients with hypertension highlights the complex interplay between gut microbiota and host metabolism in these patients. Further research is needed to elucidate the underlying mechanisms and potential causal links between these microbial markers and the development and progression of CKD and hypertension.

Functionally, the KEGG pathway and EC analysis predicted based on PICRUST2 revealed a significant association between gut dysbiosis in CKD or CKD_HTN and perturbed phenylalanine metabolism, biosynthesis of siderophore group nonribosomal peptides, as well as L-rhamnose isomerase. In our study, the metabolism of phenylalanine was significantly increased in CKD and CKD_HTN patients, which was consistent with previous research.^29^ Meanwhile, we observed a negative correlation between the abundance of *Coprococcus* genera and phenylalanine metabolism. The enhanced metabolism of phenylalanine is associated with the production of p-cresyl sulfate,^29^ a compound that has been demonstrated to exhibit renal toxicity *in vitro*,^37^^,^^38^^,^^39^ and is associated with poor outcomes in hemodialysis patients when present at high levels.^40^ Elevated plasma levels of p-cresyl sulfate in CKD patients have been shown to be toxic to endothelial cells and can lead to endothelial dysfunction and cardiovascular disorder.^41^ Moreover, a meta-analysis of 11 studies demonstrated an independent association between elevated levels of p-cresyl sulfate and increased cardiovascular events and mortality in patients with CKD.^42^ Observational studies have suggested p-cresyl sulfate as a potential biomarker for heart and kidney disease, as well as for predicting outcomes in CKD, albeit these require further validation.^43^^,^^44^ Furthermore, the metabolism of phenylalanine is widely acknowledged as a precursor to the biosynthesis of catecholamines. The aberrant state of phenylalanine metabolism in spontaneously hypertensive rats has been extensively documented.^45^ Clinical evidence from a study suggests that increased consumption of phenylalanine is associated with an elevated risk of hypertension.^46^ This can be attributed to the activation of phenylalanine metabolism, leading to heightened levels of epinephrine and norepinephrine, subsequently resulting in elevated blood pressure.^47^ In this present study, we observed a significant enhancement in the phenylalanine metabolic pathway among CKD_HTN patients, indicating its potential role as one of the underlying mechanisms contributing to hypertension development in CKD patients. Additionally, Ren et al. revealed a significant increase in the biosynthesis of siderophore group nonribosomal peptides in the gut microbial prediction function of CKD patients, which aligns with our findings.^29^ L-rhamnose isomerase, which is overexpressed in CKD and CKD_HTN patients, plays a role in fructose and mannose metabolism. This metabolic pathway is enriched in type A acute aortic dissection among hypertensive patients,^48^ providing some insight into our findings.

The gut microbiota enzyme EC:4.1.99.1, known as l-tryptophan indole-lyase, facilitates the production of indole in the intestine from dietary tryptophan and the conversion to indoxyl sulfate in the liver, a uremic toxin that stimulates progressive tubulointerstitial fibrosis and glomerular sclerosis, exacerbating kidney disorders related to CKD.^49^^,^^50^ Additionally, indoxyl sulfate has been demonstrated to induce oxidative stress in tubular cells, mesangial cells, vascular smooth muscle cells, endothelial cells, and osteoblasts as well as stimulate aortic calcification in hypertensive rats.^50^ Inhibition of l-tryptophan indole-lyase was validated as an effective approach to reduce plasma levels of indoxyl sulfate and promote renal recovery from damage caused by CKD progression.^49^^,^^51^ Therefore, targeting l-tryptophan indole-lyase holds promise as a mechanism-based strategy for preventing and treating CKD comorbid with hypertension.

The current study revealed an association between the occurrence of hypertension in CKD patients and disrupted profiles of gut microbiome, functional capacities, and related enzymes. These findings offer a potential avenue for the prevention and treatment of hypertension susceptibility in CKD, with further validation in a larger population necessary.

### Limitations of the study

There are a few limitations in the current study. This is a cross-sectional study with a limited sample size, potentially leading to underrepresentation of the population and lacking exploration of related mechanisms. In the future, it will be crucial to conduct follow-up prospective cohorts and delve into mechanistic research to effectively translate gut microbial therapy into clinical applications. Second, we acknowledge that larger studies are generally more robust in detecting subtle associations and minimizing the influence of confounding variables. Our sample size was determined based on previous studies in the field that have reported significant findings with comparable or smaller sample sizes. It is important to note that despite the limited sample size, our study identified an association between comorbid hypertension and an imbalanced gut microbiome in CKD patients, suggesting that this relationship may be detectable even in a moderately sized cohort. Future research with larger, multicenter collaborations will be crucial to further validate our findings and explore additional potential confounding factors. Third, utilizing 16S rRNA sequencing for identifying gut microbiota may introduce biases during PCR amplification steps and obtain genus data more comprehensively for identification purposes. Although this technique is suited for profiling the gut microbiota in a high-throughput manner, it could not provide the same level of resolution as whole-genome sequencing. 16S rRNA sequencing often struggles to differentiate between closely related species, which leads to an incomplete understanding of the microbial community structure at the species level. Furthermore, this method offers limited insights into the functional capabilities of the microbiome, such as gene expression, which could potentially elucidate more detailed mechanisms underlying the association between CKD, hypertension, and gut microbiota imbalance. These limitations highlight the need for further investigation utilizing more advanced sequencing technologies to gain a deeper understanding of the microbial ecosystem and its functional implications in this patient population. Diet is a critical modulator of the gut microbiome; however, controlling for dietary intake in a real-world patient population is challenging. Here, detailed dietary records were not collected as part of this study. To indirectly account for potential dietary influences, our patient population was relatively homogeneous with respect to clinical care guidelines for CKD and hypertension management, which include dietary recommendations. For medication use, all patients were under routine medical supervision, and their therapeutic regimens were made according to clinical practice guidelines, thus minimizing the impacts on our results. Future studies, ideally with prospective designs and comprehensive dietary and medication diaries, are warranted to further elucidate these complex interactions and confirm our findings.

## Resource availability

### Lead contact

Further information and requests for resources and reagents should be directed to and will be fulfilled by the lead contact, Jing Li (lijing11999@163.com).

### Materials availability

The study did not generate new unique reagents.

### Data and code availability

• Data: the datasets of this study were deposited in the National Center for Biotechnology Information under BioProject accession code PRJNA1088902 (https://www.ncbi.nlm.nih.gov/sra/PRJNA1088902).
• Code: this article does not report the original code.
• Any additional information required to reanalyze the data reported in this article is available from the lead contact upon request.

## Acknowledgments

None.

## Author contributions

Conceptualization, J.L. and Y.D.; methodology, P.W., Y.S., and K.Y.; investigation, P.W., Y.S., and K.Y.; writing—original draft, P.W., Y.S., J.L., and Y.D.; writing—review & editing, J.L. and Y.D.; resources, Q.S., S.W., J.J., H.C., and J.Z.; supervision, J.L. and Y.D.

## Declaration of interests

The authors declare no competing interests.

## STAR★Methods

### Key resources table

**Table:** 

REAGENT or RESOURCE	SOURCE	IDENTIFIER
**Deposited data**		

16S rRNA gene sequencing of the cohort	National Center for Biotechnology Information	https://www.ncbi.nlm.nih.gov/sra/PRJNA1088902

**Software and algorithms**		

QIIME2 software (version 2019.4)	QIIME	https://qiime2.org/
R software	R software	https://cran.r-project.org/

### Experimental model and study participant details

In the current study, a total of 148 participants including 54 non-CKD diagnosed controls (non-CKDs) and 94 patients diagnosed with CKD were recruited in the Department of Nephrology, Beijing Chaoyang Hospital, Capital Medical University, Beijing, China. According to the criteria of KDIGO clinical practice guidelines,^52^ subjects with kidney structural or functional abnormalities were regarded as CKD. CKD patients were further categorized into two groups: those with (CKD_HTN, n = 46) and those without (CKD, n = 48) hypertension, based on blood pressure measurements. Hypertension was defined as systolic blood pressure ≥ 140 mmHg, and/or diastolic blood pressure ≥ 90 mmHg according to the Chinese guidelines for the management of hypertension.^53^ The age of non-CKD, CKD, and CKD_HTN group is 41.00(32.75-50.00) , 37.00(30.00-47.75), and 52.50(39.75-60.75), respectively.The percentage of male in each group is 51.85%, 47.92%, and 63.00%, with no dramatic discrepancy across groups in terms of gender, which is therefore considered to exert minimal influence on the results of the study. The race of all participants is Chinese Han.

Exclusion criteria was those pregnancy, suffering from autoimmune diseases, diabetes mellitus, arrhythmia, atrial fibrillation, myocardial infarction, heart failure, stroke, peripheral artery disease, inflammatory bowel disease, cancer, and renal replacement therapy. Participants were also excluded if they had received antibiotics, probiotic treatment or undergone gastrointestinal surgeries in the preceding month.

This study was approved by the Medical Ethics Committee of Beijing Chaoyang Hospital Affiliated to Capital Medical University (Permit No. 2023-ke-730), and the protocols adhered to the principles of the Helsinki Declaration. Written informed consent was obtained from all patients prior to data collection.

### Method details

#### Sample collection

Stool sample from each participant was freshly harvested into collection containers on ice packs. These samples were then transported to the laboratory and stored at -80°C until fecal DNA extraction.

#### 16S sequencing

Total DNA was extracted from stool samples, and DNA quality was assessed by 1.2% agarose gel electrophoresis, with quantification performed using Nanodrop spectrophotometer. Following barcode sequence added, the variable region of rRNA gene was amplified through polymerase chain reaction. The V3-V4 regions of the bacterial 16S rRNA gene were amplified with primers 5’-ACTCCTACGGGAGGCAGCA-3’ (forward) and 5’-GGACTACHVGGGTWTCTAAT-3’ (reverse). Quantification of the amplified products was conducted with the Quant-iT PicoGreen dsDNA Assay Kit on Microplate reader (BioTek, FLx800). The library was prepared with Illumina TruSeq Nano DNA LT Library Prep Kit, and purified by 2% agarose gel electrophoresis. On Agilent Bioanalyzer, the library was evaluated with Agilent High Sensitivity DNA Kit, quantified on Promega QuantiFluor system with Quant-iT PicoGreen dsDNA Assay Kit, and subsequently quantified with Quant-iT PicoGreen dsDNA Assay Kit on Promega QuantiFluor system, denatured to a single strand, and subjected to paired-end sequencing on Illumina Novaseq-PE250.

#### Taxon annotation

With the raw high-throughput sequencing data acquired, the primers were removed, and we filtered the raw data and spliced the sequence with DADA2 method in QIIME2 software (version 2019.4). Under Greengenes database (Release 13.8, http://greengenes.secondgenome.com/comments), taxonomic annotation was performed with classify-sklearn methods (https://github.com/QIIME2/q2-feature-classifier).

#### Analysis of microbial diversity

Alpha-diversity parameters, including Chao1 index, Shannon, Simpson index, Pielou index and Goods coverage were calculated with QIIME2 software (version 2019.4). Richness was assessed by Chao1 and observed species indices, diversity was measured using Shannon and Simpson indices, evenness was examined by Pielou's evenness index, and Good's coverage index was used to evaluate coverage. Kruskal-Wallis rank sum test or Wilcoxon rank-sum tests was applied to perform comparison of alpha diversity indexes between groups. Rarefaction curves were obtained by randomly extracting a certain number of sequences from each sample, and calculating the total number of OTUs that the sample contain and the relative abundance of each OTU in a given series of sequencing depths with the QIIME2 software. Curves tending to be flat suggest that the sequencing data amount is progressive and reasonable, with more data facilitating to generate few novel OTUs.

According to the OTU abundance of all the samples, Bray-Curtis distance was calculated with QIIME2 software to assess the beta (between-habitat) diversity. Anosim and Permanova (permutational multivariate analysis of variance) analyses were performed using QIIME2 to assess the statistical significance of differences between groups. PCA and PCoA distance matrix were conducted by permutational multivariate analysis, and scatter plots based on Bray-Curtis distance were constructed using the vegan and ggplot2 packages in R.

#### Functional annotation

Phylogenetic Investigation of Communities by Reconstruction of Unobserved States (PICRUSt2) was used to predict the gut microbial community’s functional capabilities, and generate the KEGG (https://www.kegg.jp/) pathway and enzyme (EC) profiles. By blasting the 16S rRNA gene information to the microbial reference genome Greengene database, evolutionary tree was constructed, and the gene function of their shared ancestor was inferred. Based on the copy number of the gene family corresponding to the reference sequence in the evolutionary tree, the species with the closest sequence with the feature sequence was deduced and thus the copy number of its gene family was obtained. The gene family copy number was calculated according to the abundance of each sample characteristic sequence. The gene families were mapped to KEGG database, and the pathway information with the abundance data of metabolic pathways in each sample were obtained. Thus, the composition of the bacterial community was mapped to the database, and the gene function prediction of the gut bacteria was conducted.

### Quantification and statistics analysis

Kmeans cluster analysis and Mfuzz analysis (R, version 4.1.3; Mfuzz package, 2.54.0) were executed using the OmicStudio tools available at https://www.omicstudio.cn/tool.^54^ The relative abundance of all taxa was compared between groups with LEfSe at the Galaxy online analysis platform (http://huttenhower.sph.harvard.edu/galaxy/), and P values were obtained from Kruskal-Wallis or wilcoxon test. The corrplot package in R software was utilized to conduct Spearman’s rank correlation analysis. The q-values reported in our study were corrected for multiple testing using the Bonferroni method to ensure the validity of our findings.
