# Supplementary material for: CKD patients comorbid with hypertension are associated with imbalanced gut microbiome
Source: iScience. 2025 Jan 9;28(2):111766. doi: 10.1016/j.isci.2025.111766 (PMC11795142; doi:10.1016/j.isci.2025.111766)
Supplement: Document S1. Figures S1–S5 and Table S1 [file mmc1.pdf]

iScience, Volume *28*

Supplemental information

CKD patients comorbid with hypertension

are associated with imbalanced gut microbiome

Pan Wang, Yang Shen, Kaixin Yan, Siyuan Wang, Jie Jiao, Hongjie Chi, Jiuchang Zhong, Qianmei Sun, Ying Dong, and Jing Li

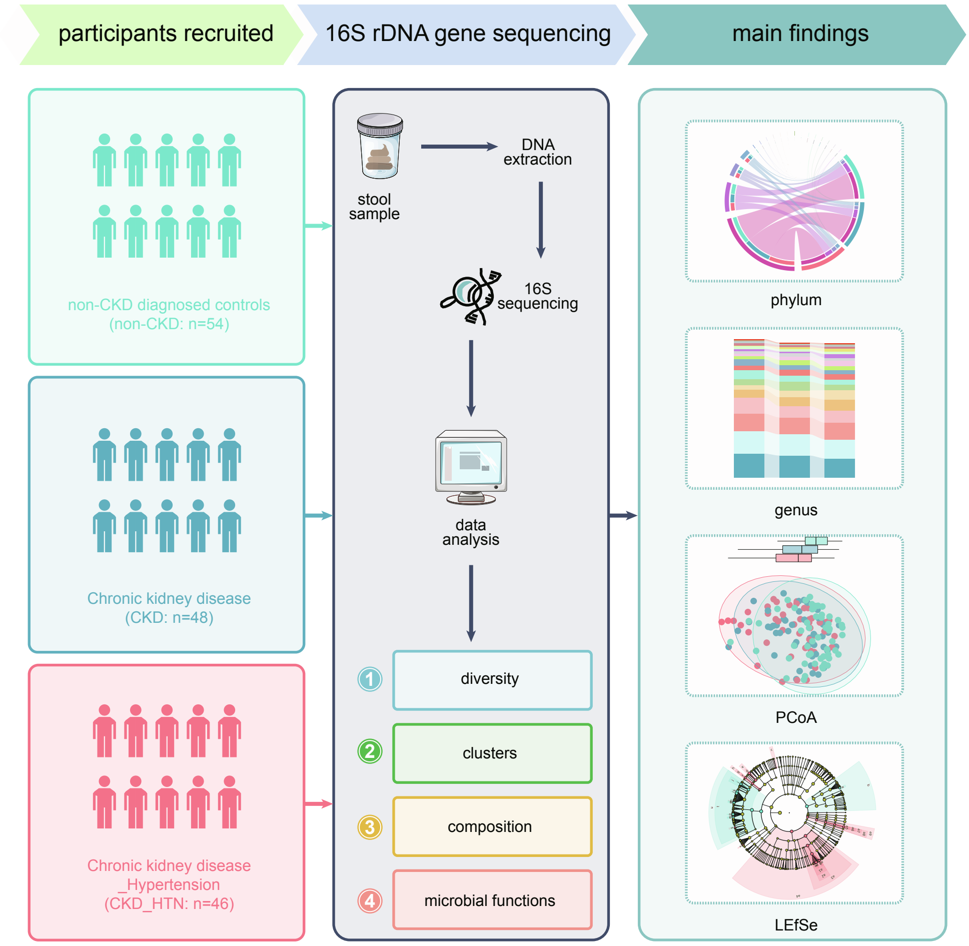

Figure S1. Study design and overview of the participants, related to Figure 1

A total of 148 individuals are enrolled in the study, including patients with a diagnosis of CKD comorbid with (CKD_HTN, n = 46) or without (CKD, n = 48) hypertension, and a group of non-CKD diagnosed individuals (non-CKD, n = 54) as compared with the patient group. Inclusion and exclusion criteria are detailed in the Methods section. Gut microbiome composition is assessed by 16S rDNA gene sequencing on stool samples. Data analysis is performed and microbial diversity, clusters, composition and function were evaluate. The main findings include taxa distributions at the phylum and genus level, diversity determined by PCoA, and microbial markers identified by LEfSe and LDA.

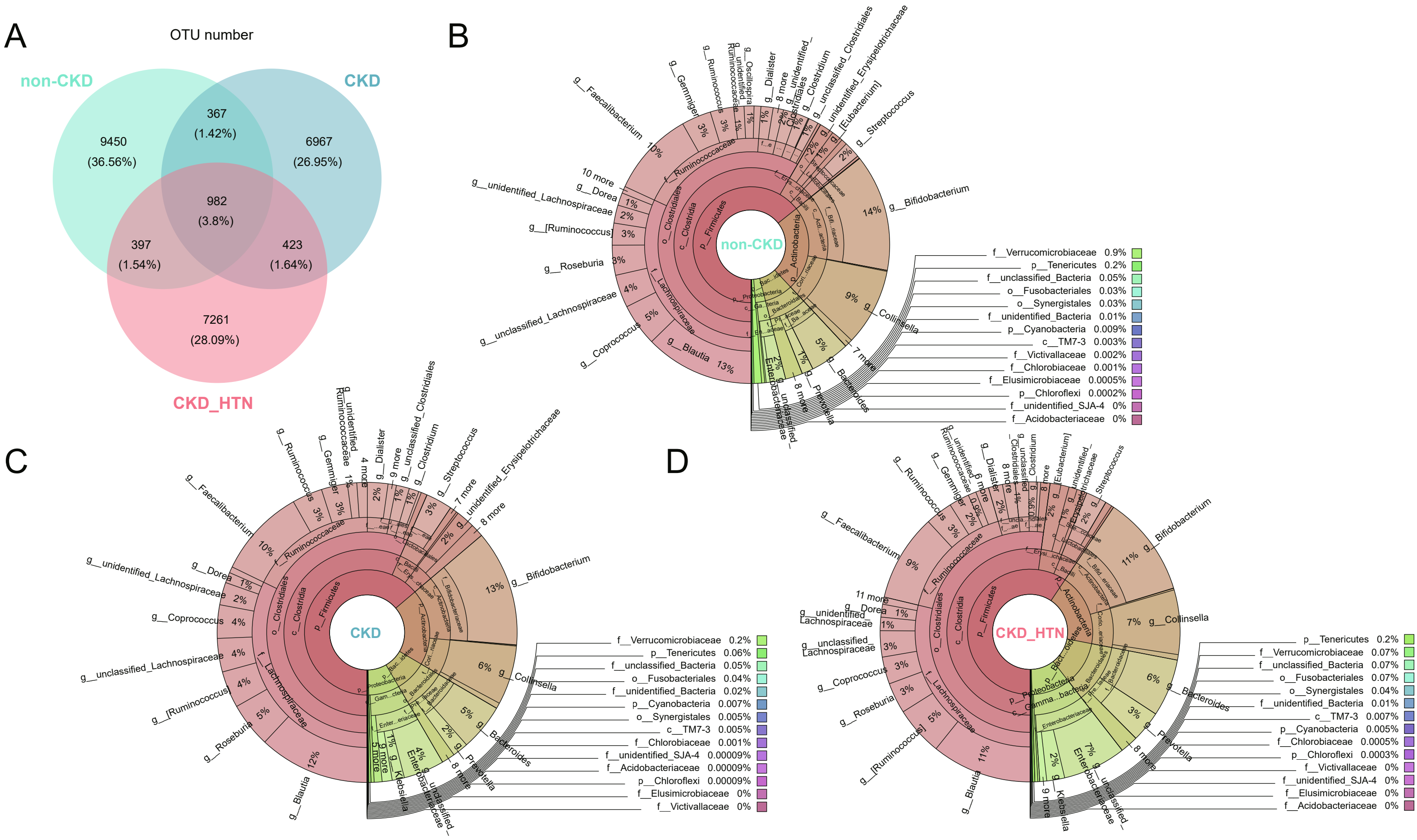

Figure S2. Overview for the gut microbes constitution of CKD patients comorbid with hypertension based on taxonomic levels, related to Figure 1

(A) Venn diagram describing the number and proportion of shared and unique OTUs iden -tified across the non-CKD, CKD and CKD_HTN groups. Overlap denotes the shared OTUs between distinct groups.

(B-D) Interactive presentations of taxonomic composition in non-CKD, CKD and CKD_HTN group are illustrated using Krona, with circles representing phyla, class, order, family, and genus taxonomic levels from inside to outside. The size of the fan denotes the relative abundance of different taxa. The taxonomic units are distinguished by colors.

A

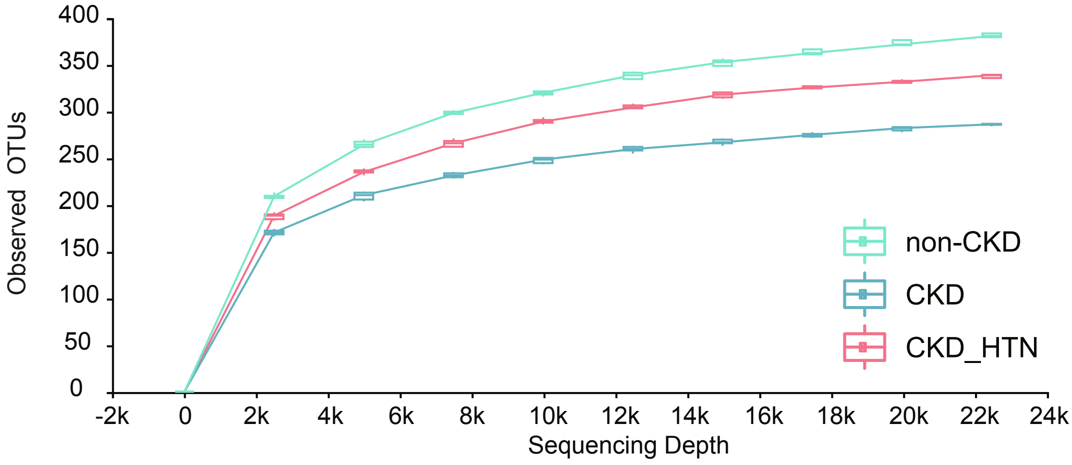

Figure S3. Rarefaction curves of distinct groups, related to Figure 2

(A) The rarefaction curve elucidates the variation trend of the number of OTUs in samples from non-CKD, CKD and CKD_HTN group with sequencing depth advanced. Boxes represent the inter quartile ranges, the inside line represent the median.

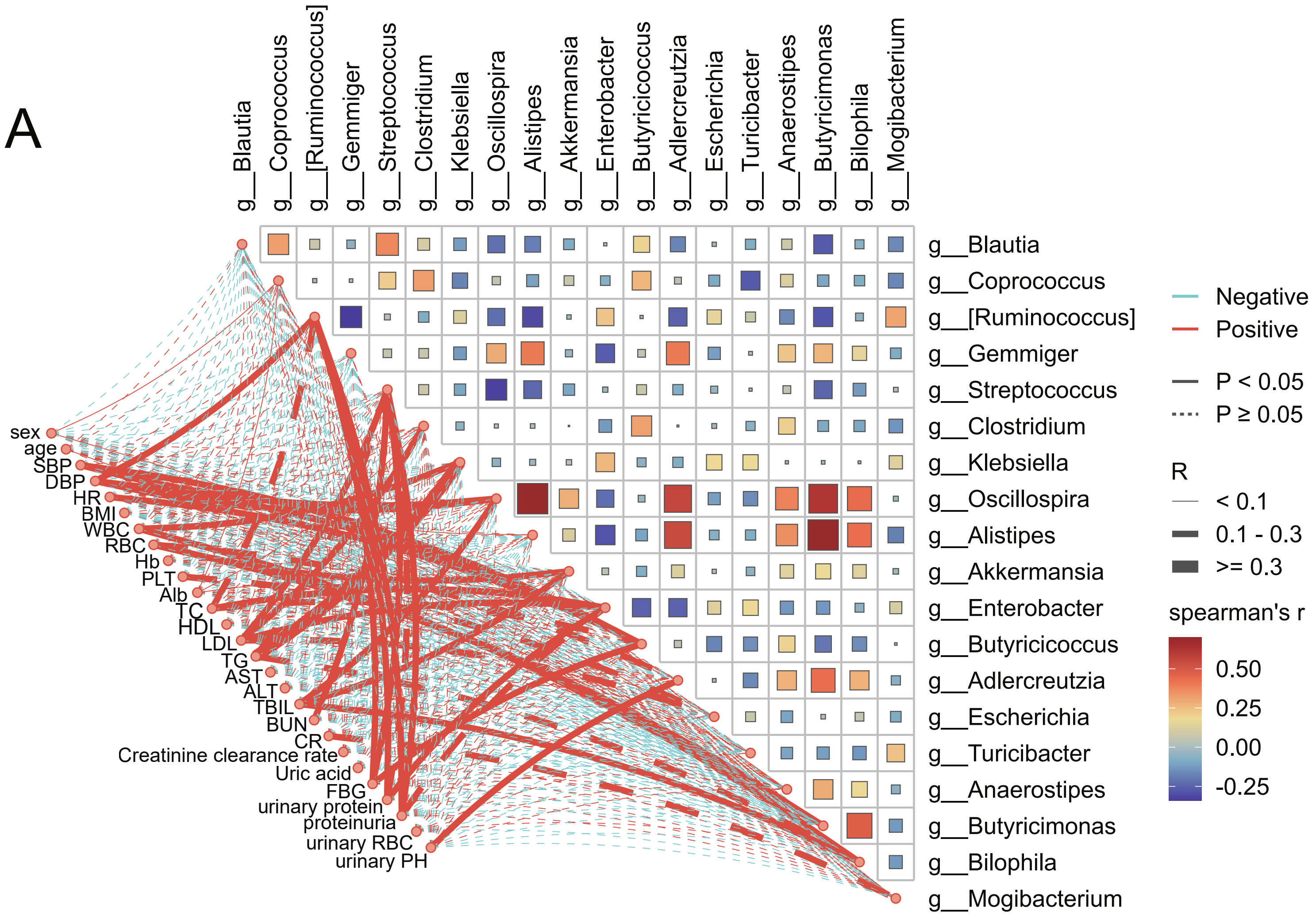

Figure S4. The association between clinical characterisitcs and the altered genera linking to CKD patients comorbid with or without hypertension, related to Figure 4

(A) Oblique triangle heatmap and network linkages show positive (red connections) and negative (blue connections) correlation between clinical indicators including sex, age, SBP (systolic blood pressure), DBP (diastolic blood pressure), HR (heart rate), BMI (body mass index), WBC (white blood cell), RBC (red blood cell), Hb (hemoglobin), PLT (blood platelet), Alb (albumin), TC (total cholesterol), HDL (high-density lipoprotein cholesterol), LDL (low-density lipoprotein cholesterol), TG (triglyceride), AST (glutamic oxaloacetic transaminase), ALT (glutamic pyruvic transaminase), TBIL (total bilirubin), BUN (blood urea nitrogen), CR (creatinine), creatinine clearance rate (creatinine clearance rate), Uric acid, FBG (fasting blood glucose), urinary protein, proteinuria, urinary RBC, urinary PH, and intestinal flora at the genus level. The degree of correlation was obtained with Spearman correlation analysis (Solid line, $P < 0.05$, dotted line, $P \geq 0.05$).

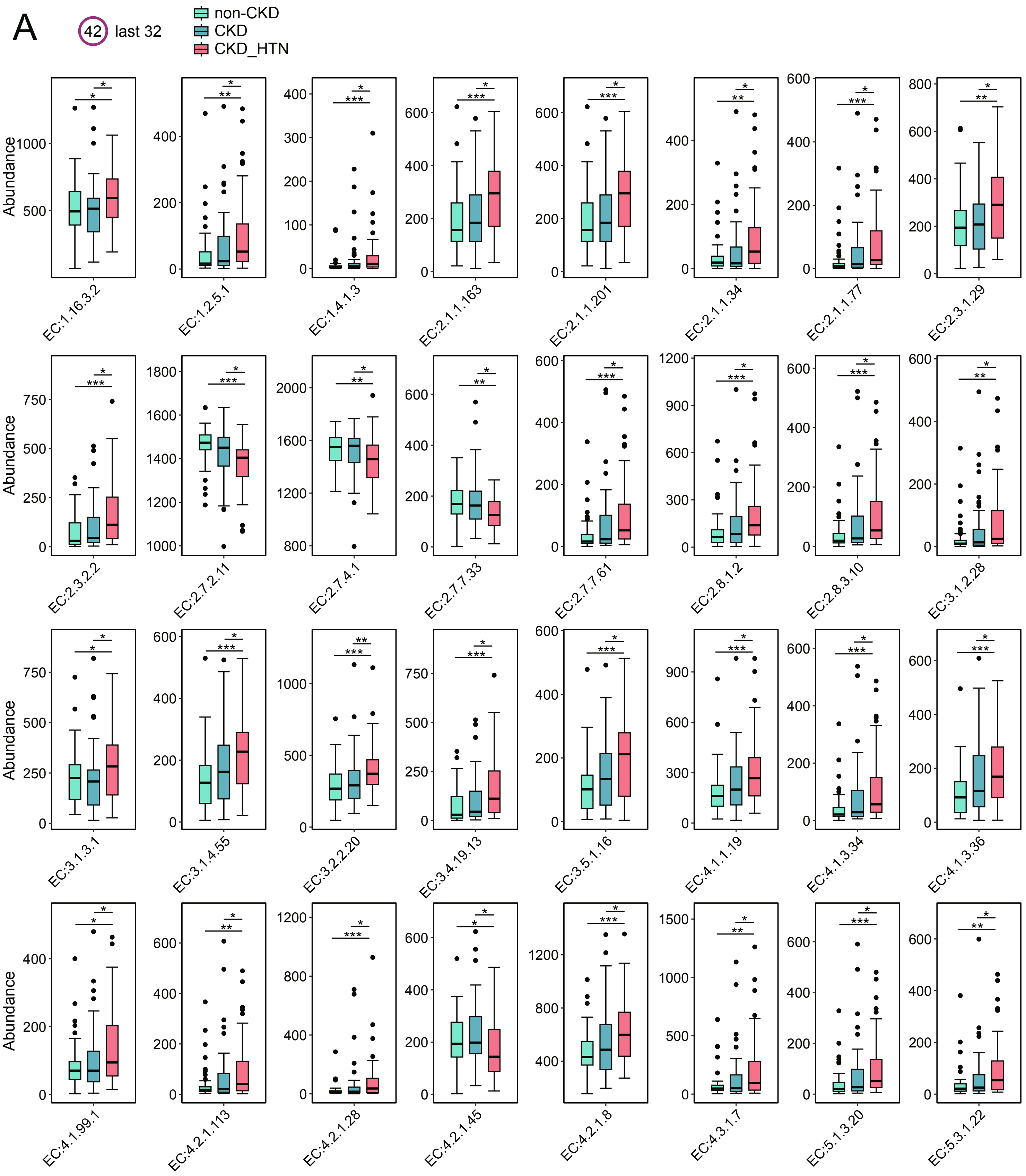

Figure S5. The core enzymes specifically shifts in CKD patients comorbid with hypertension, related to Figure 5

(A) The relative abundance of the 32 out of 42 enzymes and their distribution in the non-CKD, CKD, and CKD_HTN groups. Wilcoxon rank-sum tests (*P < 0.05, **P < 0.01, ***P < 0.001). Boxes represent the inter quartile ranges, the inside line represent the median.

Table S1. Baseline clinical characteristics of the CKD patients with or without HTN, related to Figure 1.

Characteristics	non-CKD	CKD	CKD_HTN	P_{CKD} vs. non-CKD	P_{CKD_HTN} vs. non-CKD
Number	54	48	46	NA	NA
Age, years	41.00(32.75-50.00)	37.00(30.00-47.75)	52.50(39.75-60.75)	0.207	0.001
Male (%)	51.85%	47.92%	63.00%	0.843	0.313
SBP, mmHg	116.50(110.00-125.00)	126.00(120.00-133.75)	141.00(135.00-148.00)	<0.001	<0.001
DBP, mmHg	70.00(65.75-80.00)	78.00(71.25-81.00)	86.00(79.75-92.25)	0.004	<0.001
BMI, kg/m^2	23.08(21.25-25.25)	24.34(21.78-25.78)	24.67(23.30-26.37)	0.15	0.003
TC, mmol/L	4.75(4.21-5.31)	5.86(5.20-8.27)	6.41(5.43-8.44)	<0.001	<0.001
TG, mmol/L	3.05(2.53-3.88)	3.90(2.92-5.37)	4.34(3.29-5.52)	0.002	<0.001
HDL-C, mmol/L	1.40(1.24-1.58)	1.43(1.13-1.71)	1.22(1.07-1.48)	0.82	0.034
LDL-C, mmol/L	0.96(0.65-1.38)	1.46(1.02-2.10)	1.68(1.24-2.74)	<0.001	<0.001
BUN, mmol/L	5.06(4.46-5.90)	4.61(4.11-6.32)	5.29(4.63-6.60)	0.385	0.218
Creatinine , umol/L	59.60(51.30-70.65)	67.05(53.65-82.95)	72.90(59.53-94.13)	0.065	<0.001
eGFR,ml/min	128.07(111.05-141.15)	109.02(95.04-137.06)	107.36(80.62-125.27)	0.033	<0.001
Clearance rate, ml/min	111.25(102.60-118.63)	106.58(94.71-114.94)	94.25(76.13-104.64)	0.059	<0.001
Uric acid, umol/L	312.50(247.50-380.75)	351.00(299.75-449.50)	388.00(338.25-450.25)	0.012	<0.001
Urine total protein,G/24H	0.00(0.00-0.00)	2.38(1.49-5.74)	4.16(1.63-6.34)	<0.001	<0.001
Urine specific gravity	1.02(1.02-1.03)	1.02(1.01-1.25)	1.02(1.01-1.02)	0.031	<0.001

CKD, Chronic Kidney Disease; HTN, Hypertension; SBP, Systolic Blood Pressure; DBP, Diastolic Blood Pressure; BMI, Body Mass Index; TC, Total Cholesterol; TG, Triglycerides; HDL-C, High-Density Lipoprotein Cholesterol; LDL-C, Low-Density Lipoprotein Cholesterol; BUN, Blood Urea Nitrogen; eGFR, estimated Glomerular Filtration Rate. Continuous variables are represented as median (25th and 75th quartile), and categorical variables are presented as percentages.
